# Resistance to the Beneficial Metabolic Effects and Hepatic Antioxidant Defense Actions of Fibroblast Growth Factor 21 Treatment in Growth Hormone-Overexpressing Transgenic Mice

**DOI:** 10.1155/2015/282375

**Published:** 2015-05-18

**Authors:** Ravneet K. Boparai, Oge Arum, Johanna G. Miquet, Michal M. Masternak, Andrzej Bartke, Romesh K. Khardori

**Affiliations:** ^1^Division of Geriatrics Research, Department of Internal Medicine, Southern Illinois University School of Medicine, Springfield, IL 62794-9628, USA; ^2^Division of Endocrinology, Metabolism and Molecular Medicine, Department of Internal Medicine, Southern Illinois University School of Medicine, Springfield, IL 62794-9636, USA; ^3^Department of Biochemistry, Panjab University, Chandigarh 160014, India; ^4^IQUIFIB, Facultad de Farmacia y Bioquímica, Universidad de Buenos Aires, Buenos Aires, Argentina; ^5^Burnett School of Biomedical Sciences, University of Central Florida, 6900 Lake Nona Boulevard, Orlando, FL 32827, USA; ^6^Department of Head and Neck Surgery, The Greater Poland Cancer Centre, 15 Garbary Street, 61-866 Poznan, Poland; ^7^Strelitz Diabetes Center, Eastern Virginia Medical School, Norfolk, VA 23510, USA

## Abstract

Fibroblast growth factor 21 (FGF21) modulates a diverse range of biological functions, including glucose and lipid metabolism, adaptive starvation response, and energy homeostasis, but with limited mechanistic insight. FGF21 treatment has been shown to inhibit hepatic growth hormone (GH) intracellular signaling. To evaluate GH axis involvement in FGF21 actions, transgenic mice overexpressing bovine GH were used. Expectedly, in response to FGF21 treatment control littermates showed metabolic improvements whereas GH transgenic mice resisted most of the beneficial effects of FGF21, except an attenuation of the innate hyperinsulinemia. Since FGF21 is believed to exert its effects mostly at the transcriptional level, we analyzed and observed significant upregulation in expression of various genes involved in carbohydrate and lipid metabolism, energy homeostasis, and antioxidant defense in FGF21-treated controls, but not in GH transgenics. The resistance of GH transgenic mice to FGF21-induced changes underlines the necessity of normal GH signaling for the beneficial effects of FGF21.

## 1. Introduction

The prevalence of type 2 diabetes mellitus (T2DM) worldwide was estimated to be 170 million people in 2000, and this figure is expected to increase to more than 360 million by 2030 and impose a huge public health burden [1]. The recently discovered metabolic regulator fibroblast growth factor 21 (FGF21) has been shown to exert profound antidiabetic and triglyceride-lowering effects in rodent models of diabetes and obesity, as well as in diabetic rhesus monkeys [2–4].

FGF21, a member of the FGF19 subfamily that also includes FGF19 and FGF23, lacks the conventional FGF heparin-binding domain yet exerts systemic, hormone-like effects. There is evidence that FGF21 initiates its action by interacting with a dual receptor complex of *β*-klotho and fibroblast growth factor receptor (FGFR), activating the tyrosine-kinase activity of the FGFR. The expression of both FGF21 [5, 6] and its coreceptor, *β*-klotho [7], has been demonstrated in metabolically relevant tissues such as liver, pancreas, and white adipose tissue. In deciphering the mechanistic basis for the observed effects of FGF21* in vivo*, several molecules and their corresponding pathways have been proposed as key players [8–10]. In fact, FGF21 has been shown to transduce its signal in a typical FGF manner by stimulating FGF receptor substrate (FRS2*α*) phosphorylation and activating ERK1/2 and Akt signaling pathways. Lately, it has been suggested that there is crosstalk between FGF21 and growth hormone (GH) signaling and that FGF21 can cause a state of GH resistance [11]. FGF21 overexpressing mice are reported to have elevated levels of GH and decreased levels of insulin-like growth factor-1 (IGF-1) in circulation [11]. The authors proposed that FGF21 causes GH resistance by reducing hepatic concentrations of the active form of signal transducer and activator of transcription 5 (STAT5), a major mediator of GH actions, and downregulating the expression of its target genes, including IGF-1.

GH is widely known to exert anti-insulin or diabetogenic effects on carbohydrate and lipid metabolism [12]. Hyperinsulinemia is a common feature associated with GH excess in GH overexpressing transgenic mice [13, 14] and in humans with acromegaly who often progress from GH-mediated insulin resistance to overt diabetes [15], but the cellular mechanisms underlying this form of insulin resistance remain enigmatic. While there is evidence from transgenic mouse models to show that GH excess leads to chronic activation of the IR/IRS-1/PI3K pathway, thereby reducing the extent of insulin-induced activation and resulting in decreased insulin-induced activation of key proteins including glycogen synthase [14, 16], we recently demonstrated that glucose tolerance and insulin sensitivity in transgenic mice that overexpress the bovine GH (bGH) gene are not impaired and are actually somewhat enhanced [17]. The aim of the present study was to investigate the effects of FGF21 treatment in GH overexpressing transgenic mice and to determine if FGF21 administration can rescue the hyperinsulinemic phenotype of these mice. Moreover, it has been reported that high continuous GH levels* in vivo* produce desensitization of the JAK2/STAT5 pathway of GH signaling in the liver of GH overexpressing mice [18]. We expected that as FGF21 and GH share similar signaling pathways, alterations in some of the GH signaling mediators that are involved in FGF21 signaling would hamper FGF21 action as a consequence of signaling crosstalk.

## 2. Results

### 2.1. Anatomical and Physiological Characteristics

Body weight and blood constituent parameters are summarized in Table 1. Expectedly, the body weights of GH overexpressing mice were considerably greater than their control littermates (29.9 ± 2.3 g versus 55.3 ± 0.78 g; *P* < 0.001). However, when comparing body weights before and after FGF21 treatment in chow-fed lean mice, we failed to observe a weight-lowering effect of FGF21 in mice of either phenotype. Previously, a dose dependent weight reduction effect of FGF21 has been reported in diet-induced obese mice and* ob/ob* mice [4, 19]. Moreover, FGF21 did not alter food consumption in mice of either genotype (data not shown). While a 3-fold higher concentration of insulin was observed in the GH transgenics relative to littermate controls (*P* < 0.001), fasted plasma glucose levels were only modestly (*P* = 0.034) lower in the GH transgenic mice relative to their control littermates. FGF21 treatment did not affect fasted blood glucose levels in either group of animals; however, it was able to alleviate the hyperinsulinemia in the GH transgenic mice (PBS 2.56 ± 0.80 ng/mL and FGF21 1.00 ± 0.25 ng/mL; *P* < 0.005) without causing significant change in insulin levels in normal mice (PBS 0.86 ± 0.32 ng/mL and FGF21 0.97 ± 0.16 ng/mL). Moreover, we determined the levels of *β*-hydroxybutyrate, a ketone body that is produced by the liver and serves as an alternative energy substrate peripherally when glucose is in short supply. However, as seen in Table 1, *β*-hydroxybutyrate in blood of overnight-fasted mice did not differ significantly as an effect of either genotype or treatment. In agreement with previous reports [4, 20], concentrations of plasma triglycerides and circulating NEFAs were lowered in FGF21-treated littermate control mice (*P* = 0.007 and *P* = 0.044, resp.); nonetheless, no effect was observed in the GH transgenics (Table 1).

The bovine GH overexpressing transgenics are hypercholesterolemic (*P* < 0.001) relative to control littermates, and while FGF21 treatment resulted in a modest but significant (*P* < 0.01) reduction in total cholesterol levels in control littermates, the lipid-lowering benefits of FGF21 seen in control mice did not extend to the bGH mice (Tg-PBS 119.15 ± 5.79 mg/dL and Tg-FGF21 110.40 ± 3.63 mg/dL). We also examined plasma IGF-1 levels in the mice under study and (expectedly) found a 5-fold higher concentration of IGF-1 in circulation of mice with GH excess (*P* < 0.001). While FGF21 treatment tended to cause a numerical reduction in circulating IGF-1 levels in control littermates (*P* = 0.061), it resulted in a significant (*P* = 0.032) lowering of the elevated IGF-1 levels in the GH transgenic mice (Table 1). In addition, hepatic gene expression analysis showed that FGF21 treatment resulted in significant downregulation of IGF-1 expression in normal mice (N-PBS 1.00 ± 0.15 and N-FGF21 0.46 ± 0.02; Tg-PBS 3.73 ± 0.58 and Tg-FGF21 4.07 ± 1.12) while enhancing the expression of suppressor of cytokine signaling 2 (SOCS2) in control mice but not in GH transgenic mice (N-PBS 1.00 ± 0.36 and N-FGF21 1.44 ± 0.14; Tg-PBS 6.31 ± 0.55 and Tg-FGF21 4.67 ± 0.88).

### 2.2. Intraperitoneal Glucose Tolerance Test

**Figure 1:**
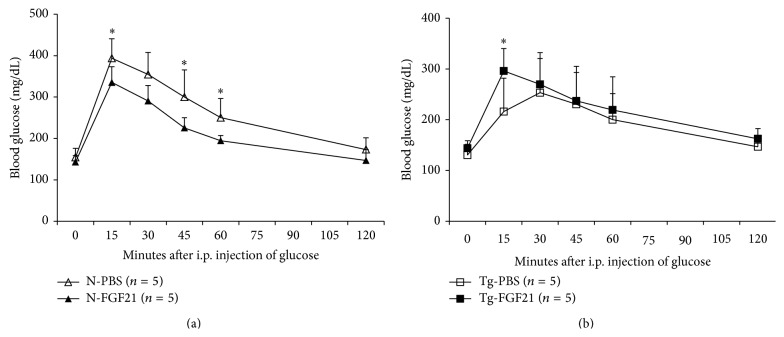
FGF21 improves glucose tolerance in normal mice. (a) Glucose tolerance testing in normal mice. (b) Glucose tolerance testing in bGH transgenic mice. Glucose levels were measured at indicated times after i.p. injection with a bolus of glucose (2 g/kg body weight). Data are mean ± SD. *P* < 0.05 versus vehicle-treated group (2-tailed, unpaired, homoscedastic Student's *t*-test).

Next, we investigated glucose disposal after a glucose challenge in normal and PEPCK-bGH transgenic mice treated with FGF21. As seen in Figure 1(a), FGF21-treated normal mice showed better glucose clearance compared to vehicle-treated animals (N-PBS versus N-FGF21; *P* = 0.016) in an intraperitoneal glucose tolerance test. At the 15-minute time-point, transgenic mice were able to clear glucose from their blood faster than control littermates and as a result showed improved glucose tolerance for the initial phase of response to the glucose challenge (*P* = 0.014). However, at later time points differences in glucose levels between PEPCK-bGH transgenics and their control littermates failed to reach significance. This seems to suggest that the elevated circulating insulin in the GH overexpressing vehicle-treated mice (Table 1) is initially able to clear the glucose load and hence only a modest increase (65%) is seen in the glucose levels of vehicle-treated transgenics at the fifteen-minute time-point in contrast to the more marked increase in blood glucose in the N-PBS group (155%). Further, as mentioned above, FGF21-treated bGH mice manifested a near normalization of the hyperinsulinemia (Table 1), which might explain the greater surge in blood glucose in the Tg-FGF21 group at 15 minutes (Figure 1(b), *P* < 0.05), relative to the Tg-vehicle-treated group, upon an exogenous glucose load. Nonetheless, exogenous FGF21 did not otherwise affect glucose disposal in mice with GH excess at later time points (Figure 1(b)).

### 2.3. Effects on Hepatic Carbohydrate Metabolism

Since FGF21 is believed to exert its effects through regulation of gene transcription [21] and the liver is the primary target for GH action, we analyzed changes in expression of genes involved in glucose metabolism in hepatic tissues of treated mice. As seen in Table 2, expression of the genes for insulin receptor (IR), insulin receptor substrate 1 (IRS1), and insulin receptor substrate 2 (IRS2) was found to be elevated in livers of littermate control mice treated with FGF21. Conversely, exogenous FGF21 failed to potentiate the expression of these constituents of the early steps of the insulin signaling pathway in livers of GH overexpressing mice (Table 2). In addition, FGF21 may have stimulated the hepatic gluconeogenic pathway, as surmised based upon observed increases in the expression of genes for the gluconeogenic enzymes, phosphoenolpyruvate carboxykinase (PEPCK), and glucose-6-phosphatase (G6Pase) in control littermates (Table 2). The expression of hepatocyte nuclear factor 4*α* (HNF4*α*), hepatocyte nuclear factor-1*α* (HNF1*α*), and peroxisome proliferator-activated receptor gamma coactivator 1 *α* (PGC1*α*), which are thought to be major mediators of the gluconeogenic process, was also induced by administration of FGF21 in normal mice without altering their expression in the GH transgenic mice (Table 2).

### 2.4. Effects on Lipid Metabolism

We examined the effects of FGF21 treatment on genes that control *β*-oxidation as well as fatty acid biosynthesis. The hepatic expression of genes involved in fatty acid oxidation was putatively promoted by FGF21 administration, as indicated by the marked increases in expression of genes involved in *β*-oxidation (Table 2). Littermate controls treated with FGF21 showed significantly increased expression of acyl-CoA oxidase 1 (ACOX1) and carnitine palmitoyltransferase 1*α* (CPT1*α*) relative to N-PBS. While bGH mice showed significantly lower mRNA levels for both ACOX1 and CPT1*α* compared to littermate controls consistent with previously published data [22], FGF21-mediated induction of genes related to hepatic fatty acid oxidation was blunted in these mice as evidenced by a lack of response for both ACOX1 and CPT1*α* in FGF21-treated bGH transgenic mice (Table 2). FGF21 had significant effects on the levels of transcripts for enzymes and transcription factors involved in the regulation of lipid metabolism. Intriguingly, it also induced expression of the transcripts for key enzymes of* de novo* lipogenesis, namely, fatty acid synthase (FASN) and acetyl CoA carboxylase (ACC), in control mice but not in GH overexpressing mice which showed baseline upregulation of the transcripts for these lipogenic enzymes (Table 2). Although investigation of corresponding enzyme activities was beyond the scope of this study, the gene expression data from our study nonetheless suggests that, in keeping with the role of FGF21 in potentiating hepatic fatty acid metabolism, it mediates parallel induction of genes related to both the lipogenic and the lipolytic pathways and hence accelerates the turnover of lipids in hepatic tissue.

We also studied the evidence for FGF21-mediated effects on uncoupling of oxidative phosphorylation as measured by changes in the mRNA for uncoupling protein 2 (UCP2), one of the mitochondrial uncoupling proteins thought to play a role in nonshivering thermogenesis and the control of mitochondria-derived reactive oxygen species (ROS). Higher expression of UCP2 was observed in littermate controls upon treatment with FGF21. While mice with GH excess showed a dramatic induction in hepatic UCP2 expression with respect to controls, exogenous FGF21 failed to alter UCP2 expression in transgenic livers (Table 2). Since 5′ adenosine monophosphate- (AMP-) activated protein kinase (AMPK) is known to be a major regulator of cellular energy homeostasis, we were interested in whether any of the observed changes in response to FGF21 administration may be mediated by AMPK. As seen in Table 2, FGF21 treatment induced AMPK expression in hepatic tissue of control littermates relative to the PBS treated controls without any effect in GH overexpressing mice.

### 2.5. FGF21 Effects on Hypothalamic Gene Expression

In view of the published evidence about the possible stimulatory effects of FGF21 on food intake, we profiled changes in the gene expression of neuropeptides involved in the control of satiety and hunger in response to FGF21 treatment. While there are some reports about increased food intake when being normalized by body weight in FGF21-treated animals and in FGF21 transgenic mice [4, 11, 19, 21], as previously mentioned, in the present study we did not observe changes in food intake in response to FGF21 administration. The hypothalamus is the site where peripheral signals and neural pathways interact to centrally regulate appetite and body weight [23]. To determine FGF21-mediated effects on central control of feeding, we looked at the mRNA levels of key hypothalamic neuropeptides such as the orexigenic neuropeptide Y (NPY), agouti-gene-related peptide (AgRP), and the anorexigenic proopiomelanocortin (POMC) in FGF21-dosed mice. As seen in Table 3, we did not observe any changes in the transcripts for any of these neuropeptides in either the control littermates or the bGH mice as a result of FGF21 administration. We also determined transcriptional changes in additional neuromodulators of feeding behavior, namely, cocaine- and amphetamine-regulated transcript (CART), orexin, leptin receptor (LEPR), and melanin concentrating hormone (MCH) and failed to observe alterations in their transcripts in response to FGF21 administration.

### 2.6. FGF21 Effects on Antioxidant Defenses

**Figure 2:**
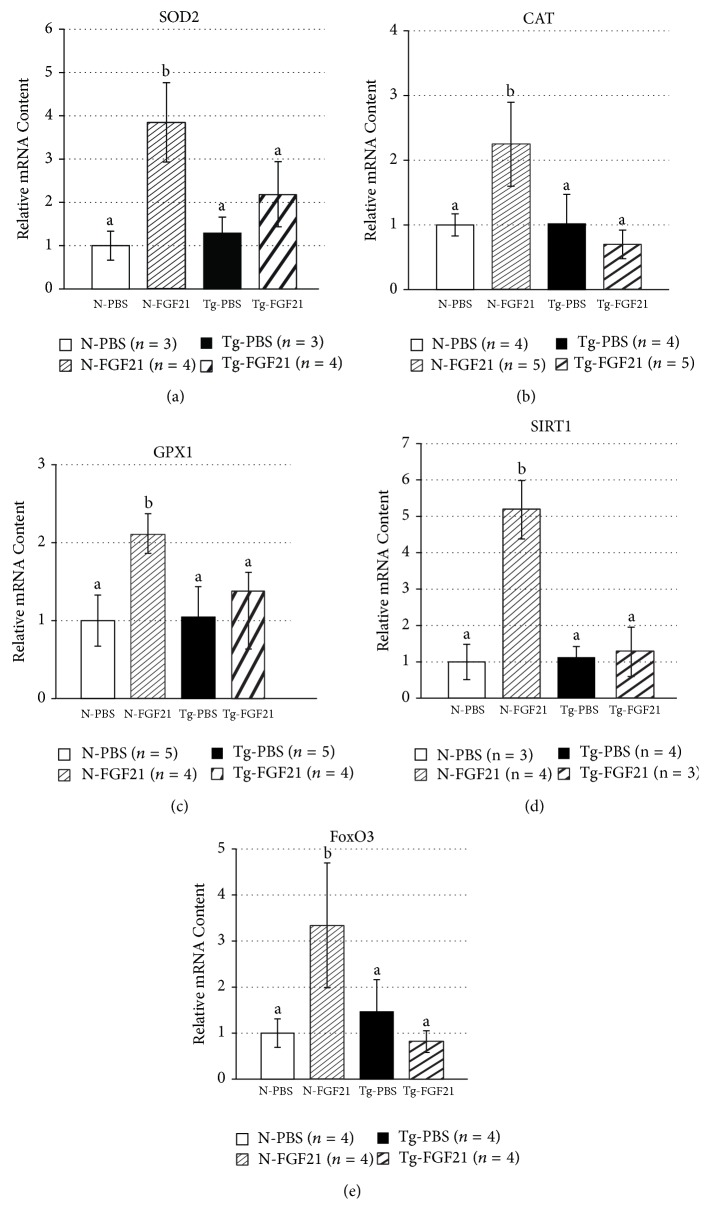
FGF21 stimulates mediators of oxidative stress resistance. RT-PCR analysis of gene expression in normal and GH transgenic mice treated with FGF21 (mean ± SEM) of the following genes: (a) superoxide dismutase 2, (b) catalase, (c) glutathione peroxidase, (d) sirtuin 1, and (e) Forkhead box class O, 3. Different superscripts denote significant difference at *P* < 0.05 (2-tailed, unpaired, homoscedastic Student's *t*-test).

Since superoxide dismutase 2 (SOD2) is a gene whose expression is regulated by insulin/IGF-1 signaling through the O family of Forkhead transcription factors (FoxO), we determined its expression in hepatic tissue of FGF21-treated mice. As seen in Figure 2, FGF21 treatment induced the expression of the gene for SOD2 in littermate control mice but not in bGH transgenic mice. Since FGF21 altered the expression of SOD2, we were interested in the supplementary effects of this hormone on antioxidant status and therefore we assessed the expression of the genes for catalase (CAT) and glutathione peroxidase (GPX1). As was the case for SOD2, FGF21 administration resulted in parallel induction for CAT and GPX1 in normal mice without significantly altering expression in transgenic mice (Figure 2). Since silent information regulator two 1 (SIRT1) and FoxO3 are believed to play a pivotal role in cellular oxidative stress resistance [24], we also examined their expression in FGF21-treated mice and found that FGF21 increased hepatic expression of both SIRT1 and FoxO3 in normal mice, yet not in bGH transgenics (Figure 2).

## 3. Discussion

One of the key novel findings of the research presented herein includes the resistance of GH overexpressing mice to the favorable effects of FGF21, pointing to the importance of normal GH signaling in mediating at least some of the wide-ranging metabolic effects of FGF21. Another important result from the present study pertains to the FGF21-mediated induction of antioxidant defenses, which might contribute to the metabolic benefits of FGF21.

FGF21 is a recently described member of the FGF19 subfamily that can act in a local and an endocrine manner to regulate glucose and lipid homeostasis. Its pharmacologic administration has been shown to improve the metabolic profile in obese and diabetic rodents and rhesus monkeys [2–4]. Although there is a preponderance of data on the broad beneficial metabolic effects of FGF21 administration, its innate physiological role and mechanism of action remain to be elucidated. In addition to being of fundamental interest to the basic biology knowledge base of endocrinology and metabolism, elucidation of FGF21's mechanism(s) of action has translational implications, insofar as drug design and drug contraindications.

Consistent with earlier publications, FGF21 administration improved glucose tolerance in response to a glucose challenge in normal mice. We also observed improvements in lipid profile, including lowering of triglycerides, free fatty acids, and total cholesterol in circulation. However, the beneficial effects of FGF21 did not extend to the bGH transgenic mice used in our study. Consistent with our previously published findings, the young-adult PEPCK-bGH transgenic mice used in our study showed somewhat better clearance, compared to control littermates, in response to an exogenous glucose load [17]. The increased musculature of the young-adult bGH mice would be expected to contribute to better glucose clearance relative to littermate controls. However, the blunted, more gradual slope of the curve in case of the bGH transgenics is noteworthy (Figure 1(b)) and could be attributed to the presence of higher circulating insulin and to defects in hepatic insulin signaling and hence impaired glucose disposal as reported previously [13]. In addition to elevated plasma insulin levels, these authors observed alterations in the early steps of the insulin signaling pathway in liver and skeletal muscle of female bGH transgenic mice.

It is of interest to note that FGF21 administration ameliorated the hyperinsulinemia in the bGH transgenic mice. The reduction in insulin levels could be attributed to enhanced insulin clearance in the liver and/or a decrease in insulin secretion. Although we do not provide direct evidence here as to which of these mechanisms is the chief contributor to lower circulating insulin, work done previously suggests that, in leptin-deficient* ob/ob* mice, FGF21 administration lowered plasma insulin levels by affecting insulin secretion; as indicated by reduced levels of amylin, a pancreatic hormone cosecreted with insulin [4]. Interestingly, expression of the FGF21 coreceptor *β*-klotho has been detected in the pancreas, which might be suggestive of local effects of FGF21 on modulating insulin secretion from mouse islets.

Since FGF21 has been purported to act by improving insulin sensitivity, the increased gene expression of molecules involved in the early steps of the insulin signaling pathway seen in the present study would concur with an insulin-sensitizing role for FGF21 (Table 2). Although we were not able to conduct direct insulin tolerance tests on these FGF21-treated mice, we assessed simple surrogate indices for insulin sensitivity/resistance such as the homeostasis model of insulin resistance (HOMA-IR) and quantitative insulin sensitivity check index (QUICKI), which all rely on fasted insulin levels. The results from these calculations suggest that, compared to their littermate controls, bGH transgenic mice are insulin-resistant, which does not agree with the insulin signaling pathway gene expression data and is further startling considering concurrent studies in which we carried out insulin tolerance tests in GH overexpressing mice of both genders and different ages and revealed comparable, if not better, insulin sensitivity in the GH transgenic mice relative to their controls [17]. While such surrogates for insulin sensitivity do show modest correlations with more direct measures of insulin sensitivity in animal models [25], in this case the particular anatomic and physiologic peculiarities of the GH transgenic mice (i.e., innate hyperinsulinemia putatively due to increased *β*-cell-to-body weight/size, as well as different body composition) might explain the divergence between surrogate measures and actual physiologic responses.

Similarly, the decreased fasting blood glucose concentration in GH transgenic mice (Table 1) should logically result (primarily, if not exclusively) from some of the traits of GH overexpressing mice (i.e., higher insulin levels (Table 1) coupled with insulin sensitivity at the level of their littermate controls [17]).

Gluconeogenic effects of FGF21, although contrary to its role in glycemic regulation, have been previously reported [26] and are generally believed to be a part of the reputed role of FGF21 in the adaptive starvation response [11, 27]. Furthermore, since both insulin and GH are major regulators of cellular metabolism and can interact functionally by signaling crosstalk, the resistance of GH transgenic mice to FGF21 may be related to GH overexpression-induced alterations in the sensitivity of various insulin and GH signaling mediators that are needed for FGF21's actions [13, 28].

FGF21 is known to regulate lipolysis and lipid oxidation in adipose tissue [29]. *β*-oxidation of fatty acids occurs in both mitochondria and peroxisomes. The first step of peroxisomal *β*-oxidation is catalyzed by ACOX1, while CPT1*α* catalyzes the transfer of long-chain fatty acids into the mitochondria and is thought to be the rate-limiting enzyme in mitochondrial fatty acid oxidation [30]. In our study, FGF21 treatment for seven days increased expression of ACOX1 and CPT1*α*, as well as the lipogenic pathway genes FASN and ACC. In addition, it also increased the transcript for AMPK in the liver, which is known to stimulate hepatic fatty acid (FA) oxidation and inhibit lipogenesis [31]. ACC phosphorylation by activated AMPK would result in disinhibition of CPT1*α* and increased fatty acid oxidation. However, it is known that full phosphorylation of ACC by AMPK results in an inhibition of ACC activity by only 50–60% [20, 32]. While a partial inhibition of ACC would redirect acetyl-CoA and malonyl-CoA flux towards fatty acid oxidation, the substantial residual ACC activity would still allow a considerable rate of lipogenesis, consistent with the increased expression of lipogenic genes that we observed (Table 2). Thus, we propose that FGF21 could orchestrate energy-dissipating futile cycling between* de novo* lipogenesis and fatty acid oxidation.

Given the large size and weight of GH overexpressing mice (Table 1), it is possible that the herein documented FGF21 resistance is secondary to excess body fat [33]. Yet, GH overexpressing mice are actually much leaner than their littermate counterparts [34]. Therefore, insofar as the prospect of adiposity-induced FGF21 resistance, the littermate controls were more likely to exhibit this confound than the GH transgenic mic, making the resistance of the GH transgenics that much more remarkable.

Our observations on hypothalamic gene expression (Table 3) contrast with a previous report that showed increases in mRNA levels for the appetite-promoting AgRP and NPY in hypothalami of FGF21-treated mice on a high-fat diet. The variability in results pertaining to not only hypothalamic gene expression but also food intake and weight loss may be explained by differences in FGF21 dose levels, duration of dosing, and the animal models. It has been previously described that the dose of FGF21 required to exert weight-lowering effects is much higher than the dose required for improvements in glucose homeostasis and insulin sensitivity [4]. Therefore, it is likely that the 0.1 mg/kg/day dose of FGF21 used in our study is insufficient to cause changes in food consumption or the central regulation of feeding behavior. Increased oxidative stress is thought to be a deleterious factor leading to insulin resistance, *β*-cell dysfunction, and ultimately Type 2 diabetes [35]. In addition, there is suggestive evidence that increased generation of ROS may impair glucose-stimulated insulin secretion and affect the expression of key *β*-cell genes [36]. In our study, FGF21-treated mice showed increased capacity for resistance to oxidative stress, as measured by the increased hepatic expression of genes for key antioxidant enzymes, while there was a lack of an effect in mice with GH excess that was consistent with observations for other metabolic genes (Figure 2). We speculate that improving antioxidant defenses may be one possible mechanism by which FGF21 improves insulin sensitivity. Additionally, increased resistance to oxidative stress is also thought to be partially beneficial, yet not necessary, for longevity [37].

Previously, Chau and others (2010) have proposed a role for FGF21 in regulating energy metabolism based on the activation of the AMPK-SIRT1-PGC1*α* pathway in adipose tissue of FGF21 administered mice [38]. In our study, FGF21 induced hepatic UCP2 gene expression and, therefore, would be expected to stimulate UCP2-dependent uncoupled mitochondrial respiration. Since PGC1*α* is known to stimulate mitochondrial biogenesis through an induction of UCP2 [39], increased UCP2 expression in FGF21-treated mice may be attributable to the FGF21-mediated induction of PGC1*α*. Moreover, increased uncoupling of oxidative phosphorylation reduces ROS production and has been postulated to be a predictor of extended lifespan, as indicated by the short-lived Ucp2^−/−^ mice [40]. Besides that, increased UCP2-dependent fatty acid oxidation appears to be another mechanism, in addition to reduced ROS generation, that can influence survival [41]. In addition, there is accumulating evidence that FoxO and sirtuin proteins (such as SIRT1), which are thought to be lifespan modulators, influence diverse physiological functions including metabolism and ROS detoxification. In fact, there is convincing evidence to show that SIRT1 complexes with FoxO3 to enhance cellular stress resistance [42], while FoxO3 is implicated in the transcriptional activation of SOD2 [43]. Upregulation of both FoxO3 and SIRT1, with FGF21 treatment, together with the FGF21-mediated induction of UCP2 and antioxidant enzymes, might be suggestive of antiaging effects of FGF21. FGF21 may thus act to improve redox metabolism with possible beneficial effects on increasing healthspan (the period of life during which an organism is able to exist and function chiefly independently and free from substantial morbidity) and/or lifespan.

To the best of our knowledge, this is the first demonstration of a role for FGF21 in boosting antioxidant defenses. Since oxidative stress is associated with chronic hyperglycemia-induced insulin resistance [44] and a decline in insulin biosynthesis and secretion [45], we are tempted to speculate that the oxidative stress resistance putatively conferred by FGF21 contributes to the beneficial metabolic actions of FGF21. Because the ability to detoxify ROS and increased oxidative stress resistance are correlated with enhanced organismal longevity in many species [46], these particular functions of FGF21 may be relevant to its ability to engender longevity [47, 48].

Li and colleagues recently reported that, in the liver, SIRT1 is necessary for fasting-induced FGF21 gene expression [49]. As our results show that FGF21 treatment is sufficient to induce hepatic SIRT1 gene expression, this combination of results from these two studies suggests a paracrine positive feedback loop in which FGF21, produced in a SIRT1-containing hepatocyte, is exocytosed and stimulates (amongst other effects) the production of SIRT1 in neighboring hepatocytes; the resulting increase in circulating FGF21 might then travel to other parts of the body to engender salubrious effects on metabolism. Although we have no data on FGF-21's effects on isolated hepatocytes or* ex vivo* liver tissues, this hypothesis, if correct, would lend support to studies concluding that FGF21 acts directly through its receptors in the liver [50], partly via SIRT1 transactivation, while still allowing for conclusions of FGF21's salutary actions on other metabolic tissues.

Finally, the resilience of the GH transgenic mice to the metabolic benefits of FGF21 treatment seems to suggest that one of the mechanisms involved in mediating the beneficial effects of FGF21 on glucose and lipid metabolism may be through GH intracellular signaling. In ongoing studies, we are investigating the effects of FGF21 on GH-resistant, GH-signaling-suppressed Laron Dwarf (*Ghr/bp* ^−/−^) mice [51]. Specifically, we are assessing the effects of FGF21 on physiological (via tolerance tests) and macromolecular measures of carbohydrate metabolism, histological and macromolecular measures of lipid/cholesterol metabolism, and metabolism as ascertained by gas (O_2_ and CO_2_) exchange-based indirect calorimetry, as well as macromolecular analyses of insulin and/or lipid signal transduction in the blood, liver, white adipose tissue, and hypothalamus in these mice lacking growth hormone hormonal signaling. The results from those studies shall clarify the issue of whether the results from the present study are directly related to the GH signaling status or to some unrelated idiosyncrasy of PEPCK-bGH mice that makes them generally meek in response to any treatment, and might make the conclusion that GH intracellular signaling is antagonistic to the endocrinologically beneficial effects of FGF21 more cogent.

## 4. Methods

### 4.1. Animals

Transgenic mice that overexpress the bovine GH gene under the control of the rat phosphoenolpyruvate carboxykinase (PEPCK) promoter have been previously described [52]. These mice had markedly accelerated postweaning growth, leading to a significant increase in body weight. Normal-sized siblings of transgenic mice were used as controls. The mice were housed three to five per cage in a room with controlled light (12 h light per day) and temperature (22 ± 2°C). The animals had free access to food (Lab Diet Formula 5001 containing a minimum of 23% protein, 4.5% fat, and a maximum of 6% crude fiber (Purina Mills Inc., St. Louis, MO)) and tap water. All experiments were performed using male mice in groups of five (3-4 months old) mice.

#### 4.1.1. *In Vivo* Protocols

The protocols used in this study were approved by the Southern Illinois University Laboratory Animal Care and Use Committee. Mice were randomly assigned to treatment or vehicle groups, based on fed glucose levels and body weight. The mice were treated with vehicle (phosphate-buffered saline (PBS)) or with recombinant human FGF21 (Tany Technogene, Rehovot, Israel) at a dose of 0.1 mg/kg/d via continuous subcutaneous infusion with microosmotic pumps (Model 1007D, Alzet, Cupertino, CA) for one week {this minimal robustly effective dosing paradigm for FGF21 was determined based on previously published dose-response data [53]}. After one week, mice were euthanized between 0900 and 1100 h by cardiac puncture under Isoflurane (Phoenix Pharmaceuticals Inc., St Joseph, MO) anesthesia. Livers were removed quickly, snap-frozen, and stored at −80°C. Blood was centrifuged (4000 ×g for 10 min. at 4°C), and plasma was stored at −80°C.

### 4.2. Intraperitoneal Glucose Tolerance Test

Intraperitoneal (i.p.) glucose tolerance tests, on mice fasted for 16 h., were performed, at 0900 h. using One Touch Ultra 2 glucometers and blood glucose testing strips (Lifescan, Inc., Milpitas, CA) to measure glucose in blood sampled from the tail vein after an i.p. injection of glucose (2 g/kg body weight/10 mL).

### 4.3. Metabolite Analysis

Glucose and *β*-hydroxybutyrate were measured in blood using the One Touch Ultra 2 glucometer and blood glucose testing strips (Lifescan, Inc., Milpitas, CA) and the Precision Xtra *β*-ketone monitor and *β*-ketone test strips (Precision Xtra, Abbott Labs, Abbott Park, IL), respectively. Serum triglycerides (GPO; Pointe Scientific Inc, Canton, MI), cholesterol (Pointe Scientific Inc, Canton, MI), and nonesterified fatty acids (NEFA's) (Wako NEFA-HR; Wako Chemicals, Richmond, VA) were measured in duplicate using enzymatic colorimetric assays. Insulin levels were determined by ultrasensitive mouse-specific enzyme-linked immunosorbent assay (ELISA) (Crystal Chem, Downers Grove, IL), while the immunoenzymometric rat/mouse insulin-like growth factor-1 (IGF-1) ELISA kit (Immunodiagnostic Systems Inc., Fountain Hills, AZ) was used for determination of circulating IGF-1.

### 4.4. Real Time RT-PCR

Total hepatic RNA was extracted using the phenol-chloroform method [54]. cDNA was obtained using the iScript cDNA Synthesis Kit (Bio-Rad Laboratories, Hercules, CA), and the relative expression of the genes was analyzed by reverse transcriptase PCR (RT-PCR) as described previously [22]. Primer sequences are available upon request. Various genes with constitutive expression including *β*2-microglobulin, GAPDH, *β*-actin, and cyclophilin A were evaluated for use as internal control, and cyclophilin A was validated and used as a housekeeping gene for normalization of RNA expression in these animals. The relative expression levels were calculated according to the formula 2^*A*−*B*^/2^*C*−*D*^ (*A* = threshold cycle (*C*_*t*_) number of the gene of interest in the first control sample, *B* = *C*_*t*_ number of the gene of interest in each sample, *C* = *C*_*t*_ number of the housekeeping gene in the first control sample, and *D* = *C*_*t*_ number of the housekeeping gene in each sample), as described previously [22]. The relative expression of the first normal sample was expressed as 1, and the relative expression of all other samples was calculated using this equation. The results from the normal group were averaged, and all the results were then divided by this average to get the fold change of expression of this gene compared with the appropriate control group (littermate control mice on PBS treatment (N-PBS)).

### 4.5. Statistical Analysis

Data were analyzed and assessed using SPSS software (SPSS Statistics 17.0, SPSS Institute Inc., Chicago, IL). Descriptive statistics of all variables were determined, including the mean and standard deviation (SD) or standard error of the mean (SEM) of each group. Data were analyzed by either Student's *t*-tests or one-way ANOVA's followed by Student Newman-Keuls* post hoc* test for pairwise comparisons, as appropriate. Data were considered significantly different when *P* < 0.05.

## Figures and Tables

**Table 1:** Body weight and plasma constituent parameters in control and GH transgenic mice.

Parameters	Normal		Transgenic	
	PBS	FGF21	PBS	FGF21
Body weight (g)	29.9 ± 2.3^a^	29.8 ± 1.0^a^	55.3 ± 0.8^b^	51.2 ± 4.0^b^
Fasting glucose (mg/dl)	143.25 ± 11.20^a^	137.75 ± 11.44^a^	117.00 ± 16.37^b^	140.50 ± 4.79^a^
Insulin (ng/ml)	0.86 ± 0.32^a^	0.97 ± 0.16^a^	2.56 ± 0.80^b^	1.00 ± 0.25^a^
*β*-Hydroxybutyrate (mmol/l)	0.43 ± 0.03	0.47 ± 0.02	0.37 ± 0.02	0.37 ± 0.02
NEFA (mmol/l)	0.51 ± 0.07^a^	0.35 ± 0.04^b^	0.57 ± 0.04^a,c^	0.69 ± 0.01^c^
Triglycerides (mmol/l)	1.21 ± 0.09^a^	0.79 ± 0.22^b^	0.98 ± 0.13^a^	1.14 ± 0.10^a^
Total cholesterol (mg/dl)	54.47 ± 5.44^a^	36.46 ± 2.79^b^	119.15 ± 5.79^c^	110.40 ± 3.63^c^
IGF-1 (ng/ml)	238.33 ± 7.93^a^	130.00 ± 21.55^a^	1112.50 ± 77.67^b^	933.33 ± 124.39^c^

Data is expressed as mean ± SD for *n* = 5 in each group. Different superscripts denote significant difference at *P* < 0.05.

**Table 2:** FGF21-induced transcriptional changes in hepatic carbohydrate and lipid metabolism.

Gene of interest	N-PBS	N-FGF21	Tg-PBS	Tg-FGF21
Insulin signaling				
IR	1 ± 0.17^a^	4.68 ± 0.91^b^	1.37 ± 0.44^a^	1.01 ± 0.12^a^
IRS-1	1 ± 0.18^a^	3.01 ± 0.63^b^	0.53 ± 0.09^a^	0.82 ± 0.15^a^
IRS-2	1 ± 0.14^a^	2.03 ± 0.21^b^	1.40 ± 0.34^a^	1.19 ± 0.22^a^

Gluconeogenesis				
PEPCK	1 ± 0.07^a^	1.82 ± 0.21^b^	0.64 ± 0.19^a^	0.54 ± .021^a^
G6Pase	1 ± 0.10^a^	2.15 ± 0.33^b^	0.59 ± 0.20^a^	0.63 ± 0.14^a^
HNF4*α*	1 ± 0.08^a^	2.34 ± 0.45^b^	0.79 ± 0.10^a^	0.79 ± 0.13^a^
HNF1*α*	1 ± 0.16^a^	3.54 ± 0.78^b^	1.14 ± 0.37^a^	1.47 ± 0.44^a^
PGC1*α*	1 ± 0.14^a^	2.37 ± 0.65^b^	0.68 ± 0.09^a^	0.88 ± 0.12^a^

Lipogenesis				
ACC	1 ± 0.12^a^	1.75 ± 0.30^b^	2.14 ± 0.36^b^	2.22 ± 0.40^b^
FASN	1 ± 0.15^a^	1.94 ± 0.32^b^	1.69 ± 0.31^b^	1.54 ± 0.26^b^

Fatty acid oxidation				
CPT1*α*	1 ± 0.19^a^	2.62 ± 0.35^b^	0.36 ± 0.11^c^	0.51 ± 0.09^c^
ACOX1	1 ± 0.11^a^	2.48 ± 0.28^b^	0.49 ± 0.10^c^	0.46 ± 0.08^c^
UCP2	1 ± 0.13^a^	1.90 ± 0.37^b^	4.15 ± 1.01^c^	4.33 ± 0.88^c^
AMPK	1 ± 0.09^a^	2.25 ± 0.52^b^	0.84 ± 0.12^a^	0.95 ± 0.10^a^

Shown is RT-PCR analysis of gene expression in normal and GH transgenic mice treated with FGF21 and vehicle (mean ± SEM, *n* = 5). Different superscripts denote significant difference at *P* < 0.05.

**Table 3:** FGF21-induced effects on genes for hypothalamic neuropeptides.

Gene of interest	N-PBS	N-FGF21	Tg-PBS	Tg-FGF21
AgRP	1 ± 0.11	1.17 ± 0.08	0.93 ± 0.21	0.86 ± 0.10
POMC	1 ± 0.17	1.22 ± 0.09	1.04 ± 0.09	1.32 ± 0.14
CART	1 ± 0.01	1.01 ± 0.08	0.92 ± 0.11	1.08 ± 0.03
NPY	1 ± 0.06	1.04 ± 0.02	1.14 ± 0.13	1.17 ± 0.09
MCH	1 ± 0.12	0.95 ± 0.08	1.12 ± 0.04	1.26 ± 0.09
Orexin	1 ± 0.10	1.17 ± 0.12	1.09 ± 0.05	1.21 ± 0.14
LEPR	1 ± 0.12	1.21 ± 0.07	1.49 ± 0.29	1.77 ± 0.33

Shown is RT-PCR analysis of gene expression in normal and GH transgenic mice treated with FGF21 and vehicle (mean ± SEM, *n* = 5).
